# Thermodynamic Insight into Donor–Acceptor Cyclopropane Reactions with 1-Methylindole

**DOI:** 10.3390/molecules31214023

**Published:** 2026-11-08

**Authors:** Charles Biaku, Boyi Zhang, Parker Maloney, Malik Bell, Kristine Nolin, Carol A. Parish

**Affiliations:** Department of Chemistry, Gottwald Center for the Sciences, University of Richmond, 28 Westhampton Way, Richmond, VA 23173, USAknolin@richmond.edu (K.N.)

**Keywords:** donor–acceptor cyclopropane, methylindole, density functional theory, mechanism

## Abstract

Computational and experimental approaches were used to study the reaction of dimethyl 2-phenylcyclopropane-1,1-dicarboxylate with 1-methylindole. In these reactions, the cyclopropane ring is activated for heterolytic cleavage by the electron-withdrawing properties of the methyl esters at C1 and the electron-donating behavior of the phenyl group at C2. The reaction with 1-methylindole produces a diastereomeric zwitterionic intermediate that can either abstract hydrogen from the indole C4 to form a Friedel–Crafts addition product or proceed via electrophilic addition and intramolecular cycloaddition to form a cyclopenta-fused indole. Density functional theory calculations with the B3LYP functional and the 6-31G** basis set were used, in a variety of solvent environments, to determine the energies and conformations of the zwitterionic intermediate as well as the Friedel–Crafts and annulated products. Three stable conformations were identified for each diastereomeric zwitterionic intermediate. The lowest energy conformer contained a geometry pre-optimized for hydrogen abstraction from C4 leading to the Friedel–Crafts product. The second most stable zwitterionic conformer placed the C1 enolate within 3.7 Å of C5, facilitating the formation of the annulation product. Barriers between all species were determined. The Friedel–Crafts product was 5–11 kcal/mol more stable than the annulation product, depending on the solvent environment. To verify these findings, we monitored the disappearance of reactant and formation of products using NMR. Initially, annulation proceeded faster than Friedel–Crafts product formation; however, as the reaction proceeded, a full conversion of cyclopropyl reactant to Friedel–Crafts product was observed, in agreement with the computational results suggesting that the Friedel–Crafts product was enthalpically favored.

## 1. Introduction

Donor–acceptor (DA) cyclopropanes are a privileged class of compounds in organic synthesis. Their vicinal electron-withdrawing (EWG) and electron-donating groups (EDG) activate the cyclopropyl C–C bond toward cleavage in a variety of reactions [1,2,3,4,5]. The study of DA cyclopropanes has increased dramatically in the last decade because of their synthetic versatility [6,7,8,9,10]. Various combinations of donor and acceptor groups can be incorporated onto the cyclopropane ring, enabling a wide range of possible reactions [3,11,12,13]. Nucleophilic ring-opening, rearrangement, and cycloaddition reactions have all been studied using DA cyclopropanes [5]. In this study, we investigate reaction pathways for the Friedel–Crafts addition of 1-methylindole to DA cyclopropanes.

**Figure 1:**
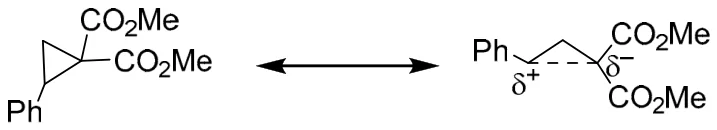
The effects of EDGs (phenyl) and EWGs (CO_2_Me) on the C-C bond in cyclopropane.

The cyclopropane in this study bears geminal diester substituents. These two esters are electron-withdrawing groups (acceptors), which pull electron density from the cyclopropyl C–C bond. The partial withdrawing nature of these groups causes a polarization of the sigma bond. The developing positive charge is stabilized by the phenyl ring, and the donor group (EDG). The donor and acceptor groups work synergistically to activate the C-C bond (Figure 1) [2,4]. The zwitterionic character of the DA cyclopropane will vary as the strength of the donor/acceptor groups varies.

**Scheme 1:**
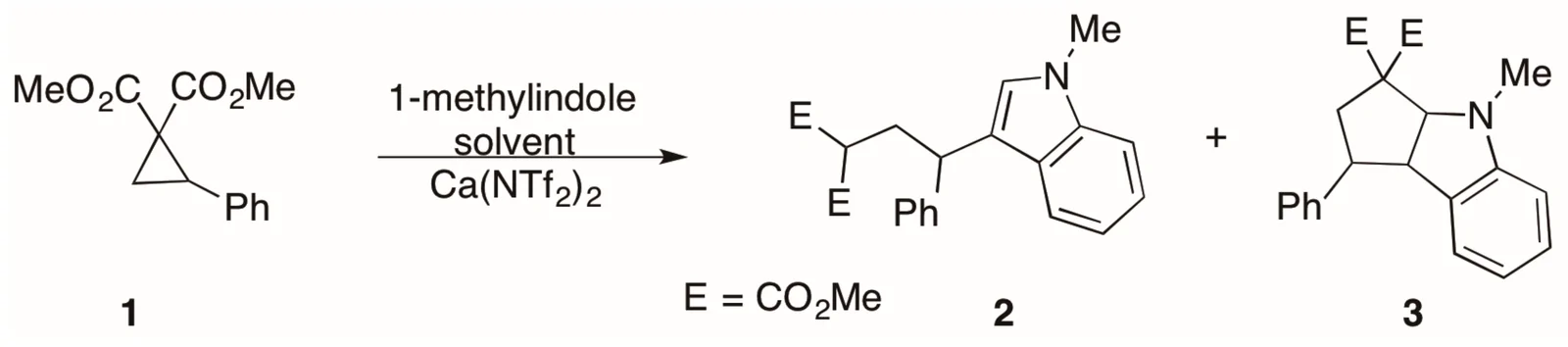
The reaction of 1,1-cyclopropanediester with 1-methylindole facilitated by a calcium-based catalyst.

In a previous report, Dulin et al. described the reaction of 1,1-cyclopropanediesters with 1-methylindole, facilitated by a calcium-based catalyst [14]. Their studies showed that 1,1-cyclopropanediester derivative **1** reacted with 1-methyindole to form Friedel–Crafts addition product **2** in the presence of catalytic amounts of Ca(NTf_2_)_2_. Also formed under the reaction conditions, albeit reversibly, was annulation product **3** (Scheme 1).

Kerr et al. described the formation of Friedel–Crafts products from 1,1-cyclopropandiesters and various alkylindoles when ytterbium triflate was used as the catalyst [15,16]. Venkatesh et al. have explored conditions under which donor–acceptor cyclopropanes react with indoles to form annulation products similar to **3**. They found that a number of these reactions proceeded in a stereoselective manner and suggested a reaction mechanism that included the formation of the DA cyclopropane zwitterion intermediate [16].

Density functional theory (DFT) studies by Wanapun et al. have shown that concerted and stepwise pathways for reactions between DA cyclopropanes and nitrones, via zwitterionic intermediates, are energetically similar [17]. In polar solvents, a stepwise mechanism is favored by stabilizing this intermediate. In a related study, Zhang et al. used DFT to examine the reaction of DA cyclopropanes and benzaldehyde. They concluded that the reaction proceeded via a stepwise mechanism that included an S_N_2-like back side attack of the carbonyl oxygen on the cyclopropyl ring resulting in the cleavage of the cyclopropyl C–C bond [18].

**Scheme 2:**
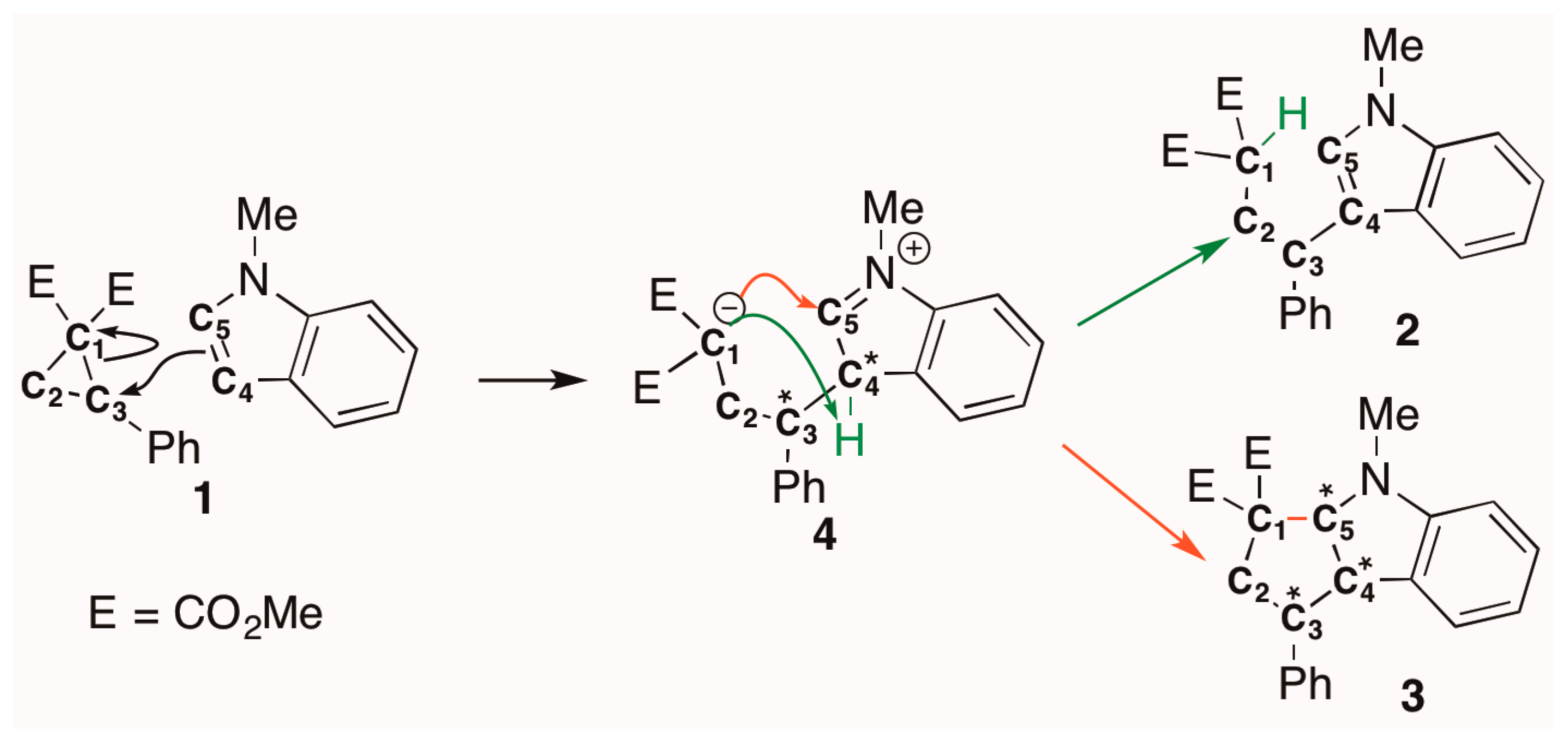
Proposed reaction mechanism. Green and red arrows highlight the Friedel Crafts and annulation pathways, respectively.

**Figure 2:**
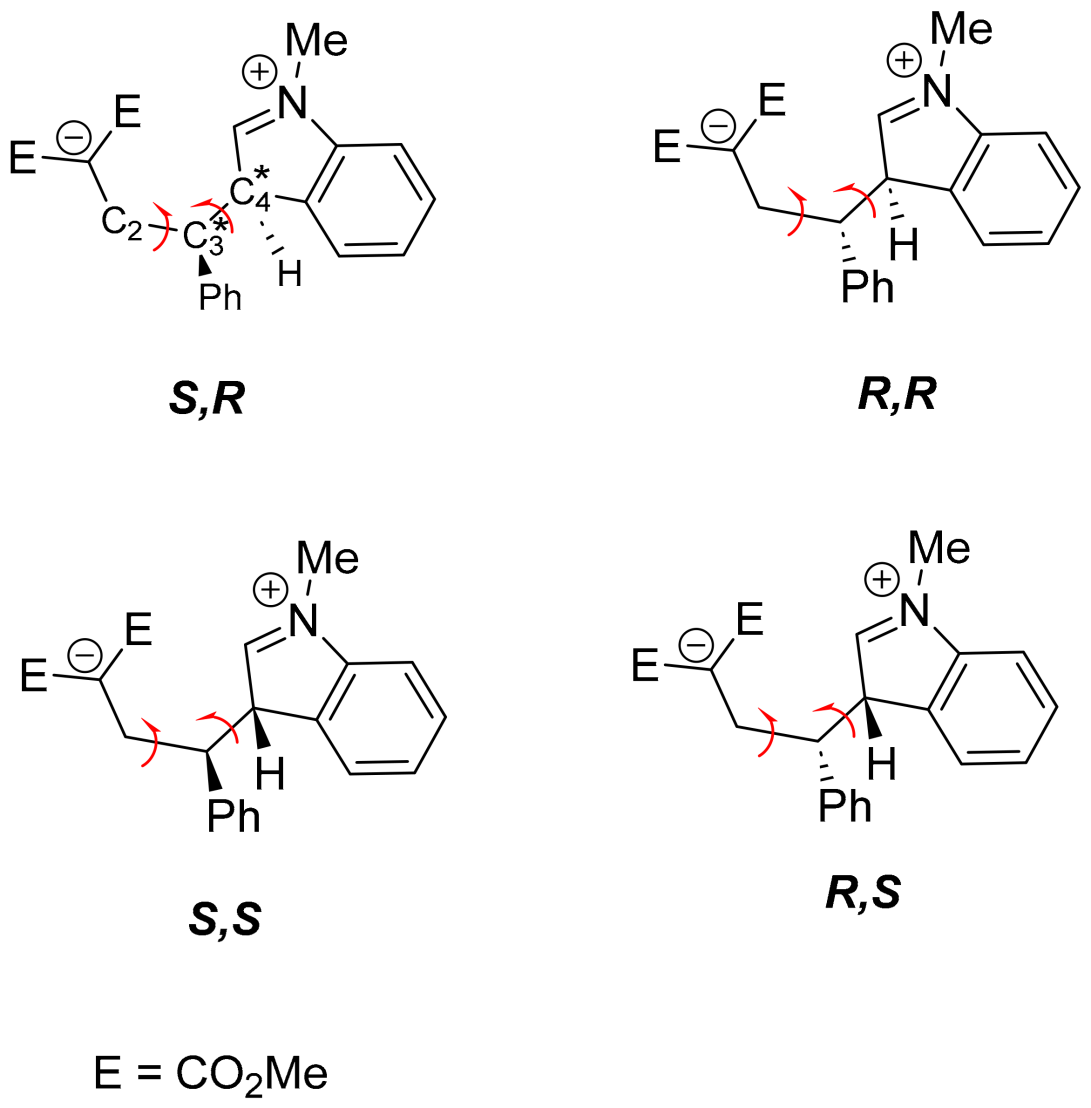
Four stereoisomers of reactive intermediate **4**. Red arrows highlight the two torsional degrees of freedom that were conformationally sampled.

In our previous experimental study [14], we explored a variety of calcium-based catalysts for the reaction of cyclopropane with methylindole (Scheme 1). It is a challenge to experimentally characterize reaction intermediates as they are often transient and difficult to isolate. In this study, DFT methods were used, in conjunction with ^1^H NMR time studies, to investigate the conformational dynamics of the zwitterionic intermediate as well as the thermodynamics for the reaction between dimethyl 2-phenylcyclopropane-1,1-dicarboxylate **1** with 1-methylindole. We are purposely performing this study in the absence of a calcium-based catalyst in order to determine thermodynamic baseline behavior, i.e., the uncatalyzed reactivity. We examine the likelihood that the reaction leading to the formation of Friedel–Crafts product **2** and annulation product **3** proceeds through putative reactive intermediate **4** (Scheme 2). We are especially interested in the stability of the zwitterionic intermediate as well as the products, in the absence of a catalyst. The presence of two chiral centers in **4** means that there may be up to four stable stereoisomers of the reactive intermediate (Figure 2). The structure and energetics of all of these options were explored in various solvent environments.

## 2. Results and Discussion

### 2.1. Conformational Dynamics of Reactive Intermediate ***4***

**Figure 3:**
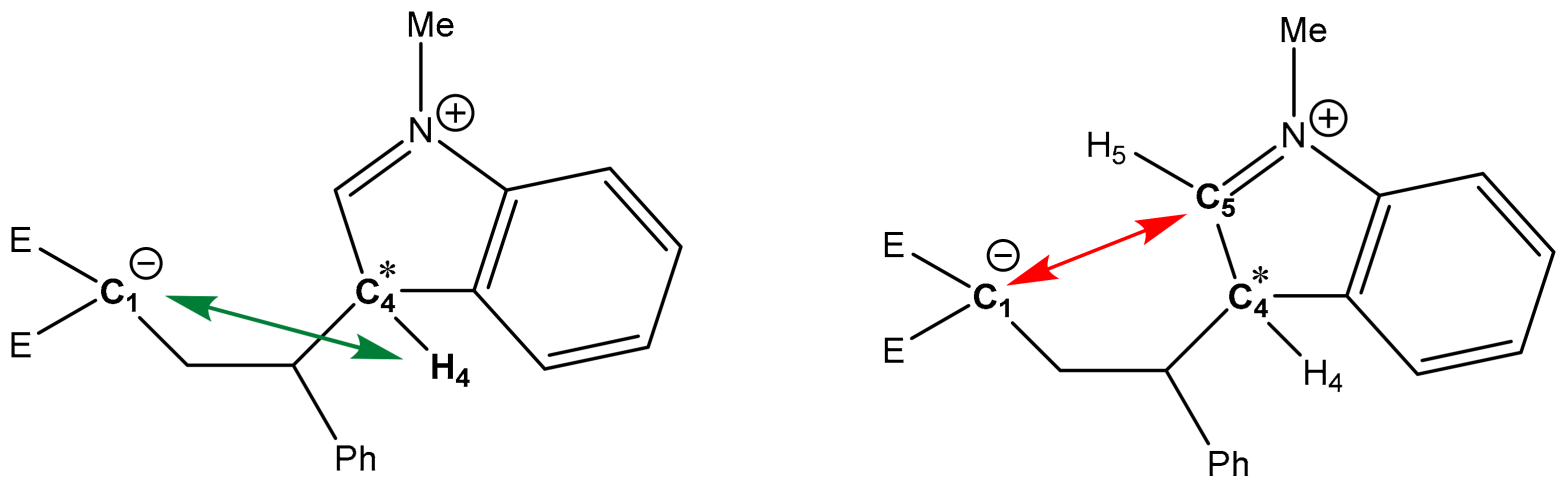
Important distances in intermediate conformer **4**. LHS: **C1** enolate abstracts a hydrogen atom from the **C4** carbon, forming Friedel–Crafts product **2**. RHS: **C1** enolate forms a bond with **C5** generating annulation product **3**.

For the reaction of 1,1-cyclopropanediester **1** with 1-methylindole, a nucleophilic attack of **C4** on the benzylic cyclopropyl carbon atom, **C3,** results in the cleavage of the **C1**–**C3** bond and the formation of **4**. This zwitterionic intermediate can further react via two pathways. First, the **C1** enolate abstracts hydrogen from the **C4** carbon, forming Friedel–Crafts product **2**. This pathway depends on the proximity of the **C1** anion to hydrogen on **C4** as measured by the **C1**_anion_–**H4** distance (Figure 3). In the second pathway, **C1** will form a bond with **C5** to create annulation product **3**. The formation of this product will depend on the **C1_anion_**–**C5** distance (Figure 3). The **C1_anion_**–**H4** and **C1_anion_**–**C5** distances have been examined with respect to the diastereomeric conformations identified for reactive intermediate **4** (Table 1).

In all solvents examined (CH_2_Cl_2_, THF, CHCl_3_ and toluene), three different conformational isomers resulted from the search of torsional space for reactive intermediate **4** (Table 1). Each conformer (IC1–IC3) was contained within a distinct minimum energy well on the quantum potential energy surface, as confirmed by vibrational frequency analysis. The conformer represented by intermediate conformer group 1 (IC1) has an optimized C2-C3 torsion angle of ~±50°. Intermediate conformer group 2 (IC2) has optimized torsion angles of ~ ±67° and intermediate conformer group 3 (IC3) has optimized angles of ~±170°. Table 1 shows representative conformer structures using results for the *RS*/*SR* enantiomer of **4** in DCM.

All of the stereoisomers of **4** adopt IC1–IC3 conformations in all solvents (Table 2). IC1 RS/SR enantiomers are the lowest energy structures across all solvents, typically lying 2–3 kcal/mol lower than their diastereomeric partners IC1 RR/SS and up to 11 kcal/mol lower in energy than other conformers. The IC1 C2–C3 torsion angle of ±50° orients **C1_anion_** close to **H4**, with distances ranging from 2.08 Å for RR/SS in toluene, to 2.38 Å for RS/SR in DCM. Given these short distances, and the overwhelming stability of the IC1 conformer, we would predict that the reaction shown in Scheme 2 would be enthalpically most likely to produce Friedel–Crafts product **2**.

For IC2 structures, the torsion angle of ~ ±63 degrees about the C2–C3 bond results in **C1** pointing toward **C5** and away from hydrogen on **C4** resulting in some of the shortest **C1_anion_**–**C5** distances in the IC2 structures (see representative IC2 structure in Table 1). This suggests that IC2 is enthalpically the most probable conformation to undergo the annulation reaction to form **3**. However, the higher energy of this intermediate conformer, relative to IC1, suggests that while both the Friedel–Crafts **2** and annulation **3** products are possible, the annulation population will likely be the smaller component of the product mixture.

The C2–C3 torsion angle of ~±180 degrees in IC3 structures results in the **C1_anion_** pointing away from both **H4** and **C5**. The large distances between the reactive centers of IC3 makes it less likely to optimize to either the **2** or **3** products. IC3 conformers are also the highest energy minima, lying almost 5 to more than 11 kcal/mol higher in energy than the IC1 conformers, suggesting a fleeting population of these high energy conformations and a relatively small contribution to the overall product ensemble. The reduced stability in IC3 is likely attributable to a less effective intramolecular stabilization of the zwitterionic charges due to the greater **C1_anion_**–**H4** and **C1_anion_**–**C5** separations, relative to the shorter distances in the more compact and lower-energy IC1 and IC2 structures. The **C1_anion_**–**H4** and **C1_anion_**–**C5** distances for all stereoisomers in all solvents are listed in Table 2. A graphical comparison of the energies and **C1_anion_**–**H4** and **C1_anion_**–**C5** intramolecular distances, of all minimum energy conformations of **4** in a variety of solvents, is summarized in Figure S1.

The relative enthalpies in Table 2 show that the intermediate conformations of **4** are most stable in DCM followed by THF. Given the zwitterionic nature of **4**, enhanced stability in more polar solvents is expected. The intermediate conformations were less stable in chloroform and least stable in toluene. These results are in agreement with solvent effects reported by Wanapun et al. [19].

### 2.2. Product Energetics

The relative enthalpies of Friedel–Crafts product **2** and the diastereomeric *cis* (RR/SS) and *trans* (RS/SR) isomers of annulation product **3** are summarized in Table 3. The *cis* and *trans* annulation products, **3a** and **3b**, respectively, are 4.2–5.3 kcal/mol less stable than the Friedel–Crafts product, with energetic values dependent on solvent environment.

### 2.3. Reaction Barriers

The results described above provide detailed information regarding the thermodynamic conformational preferences of the reactants, reactive intermediates and products. To better understand the kinetics associated with this reaction, we also identified transition states associated with the reaction in dichloromethane (DCM) and THF. These solvents were chosen for the barrier study as these were the solvents in which the reactive intermediates were most stable. We also focused our transition state study using the lowest energy intermediate RS/SR IC1, which led to FC product **2**, and IC2, which led to annulation product **3** (Table 2).

**Figure 4:**
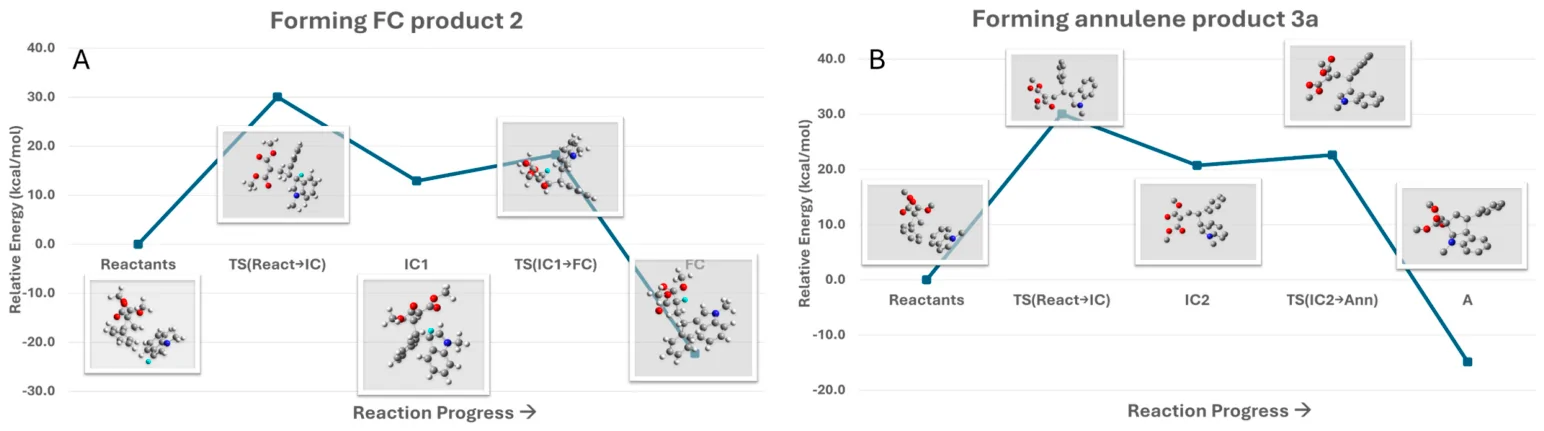
Enthalpies for the reaction of 1,1-cyclopropanediester (**1**) with 1-methylindole in dichloromethane to form (**A**) the Freidel Crafts (**2**) and (**B**) annulation products (**3a**). Stationary points are computed with B3LYP/6-31G** in IEFPCM solvent. Hydrogen atoms have been retained in the structures shown in A as the second transition state involves H migration; however, hydrogens have been removed from the structures in B for ease of viewing.

The effective barrier for reaction of 1,1-cyclopropanediester **1** with 1-methylindole to form the RS/SR diastereomer of **4** is very similar in DCM and THF; 30.0 and 30.6 kcal/mol, relative to the reactants, respectively. The energies relative to the separated reactants are comparable because they are essentially the same transition state (TS) structure, albeit in different solvents, with the C4 atom on the pyrrole ring of methylindole 2.12 Å from the C3 atom on the cyclopropane ring (for atom numbering, see Scheme 2.) The animation of the -376 cm^−1^ vibrational frequency shows this distance decreasing to form a sigma bond between C4 and C3, leading to the reactive intermediate **4** as proposed in Scheme 2. Once formed, **4** can adopt three different conformations, as discussed above. In the surfaces shown in Figure 4 and Table 4, we focus on the two lowest-energy conformations of **4** (IC1 and IC2), which convert to **2** and **3**, respectively. It is this second set of barriers that distinguish between the Friedel–Crafts and annulation pathways. The barrier for conversion from IC1 to the lowest-energy FC product **2** lies at 12.9 and 13.6 kcal/mol in DCM and THF, respectively. The animation of the negative frequency associated with this structure (-499 and -452 cm^−1^ in DCM and THF, respectively) shows the hydrogen atom bonded to C4 in IC1 migrating to the anionic C1 to form minimum energy structure **2** (Figure S2). We also follow the second-lowest energy reactive intermediate (IC2), which leads to a transition state that lies 18.2 and 18.8 kcal/mol higher in energy than reactants, in DCM and THF, respectively. The animation of the negative frequency associated with this TS structure shows a shortening of the distance between the anionic C1 on the cyclopropyl moiety and the C5 atom on methylindole, leading to annulene product **3a**. The distance between C1 and C5 in this TS structure is 2.7 Å. Figure 4 illustrates the overall reaction enthalpies using values in DCM. As can be seen in Table 4, the enthalpies in THF are comparable.

### 2.4. Experimental Verification by NMR

Previously, when the reaction was run at 50 °C in 2-MeTHF, we saw the initial formation of annulation product **3**, followed by conversion to Friedel–Crafts product **2** [14]. After 21 h, the reaction achieved 100% conversion with a >99:1 selectivity for the FC product. The computational results suggest that IC1, the most thermodynamically stable reactive intermediate, adopts a conformation that favors the formation of **2**. Once formed, **2** is more stable than annulation product **3**. We sought to experimentally confirm these thermodynamic preferences by using NMR to monitor the reaction, in situ, until completion. To aid in the identification of the various species, we utilized the gauge-independent atomic orbital (GIAO) approach with the B3LYP/6-31G** level of theory to compute the chemical shifts in the H4 and H5 protons (Table 5).

Calculations were performed on all unique, optimized, intermediate conformers of **4** as well as products **2**, **3a** and **3b**, to determine the ^1^H NMR chemical shifts in THF. As can be seen in Table 5, the **H5** in **4** is shifted downfield for all conformers. This is consistent with a partial positive charge on **C5**. Upon the conversion of **4** to **2**, **H5** shifts upfield to 7.23 ppm. A larger shift occurs upon the addition of **C1** to **C5**. The annulation of **4** to yield **3** places **H5** on the newly formed sp^3^ carbon, which is consistent with the simulated chemical shift. Additionally, **H4** is shifted upfield but to a lesser magnitude than **H5** for most conformers. This is attributed to proximity of H4 to the induced magnetic field of the phenyl ring. Given the upfield shift in the products, it is believed that **H4** is located within this field, inducing anisotropic magnetic shielding.

**Figure 5:**
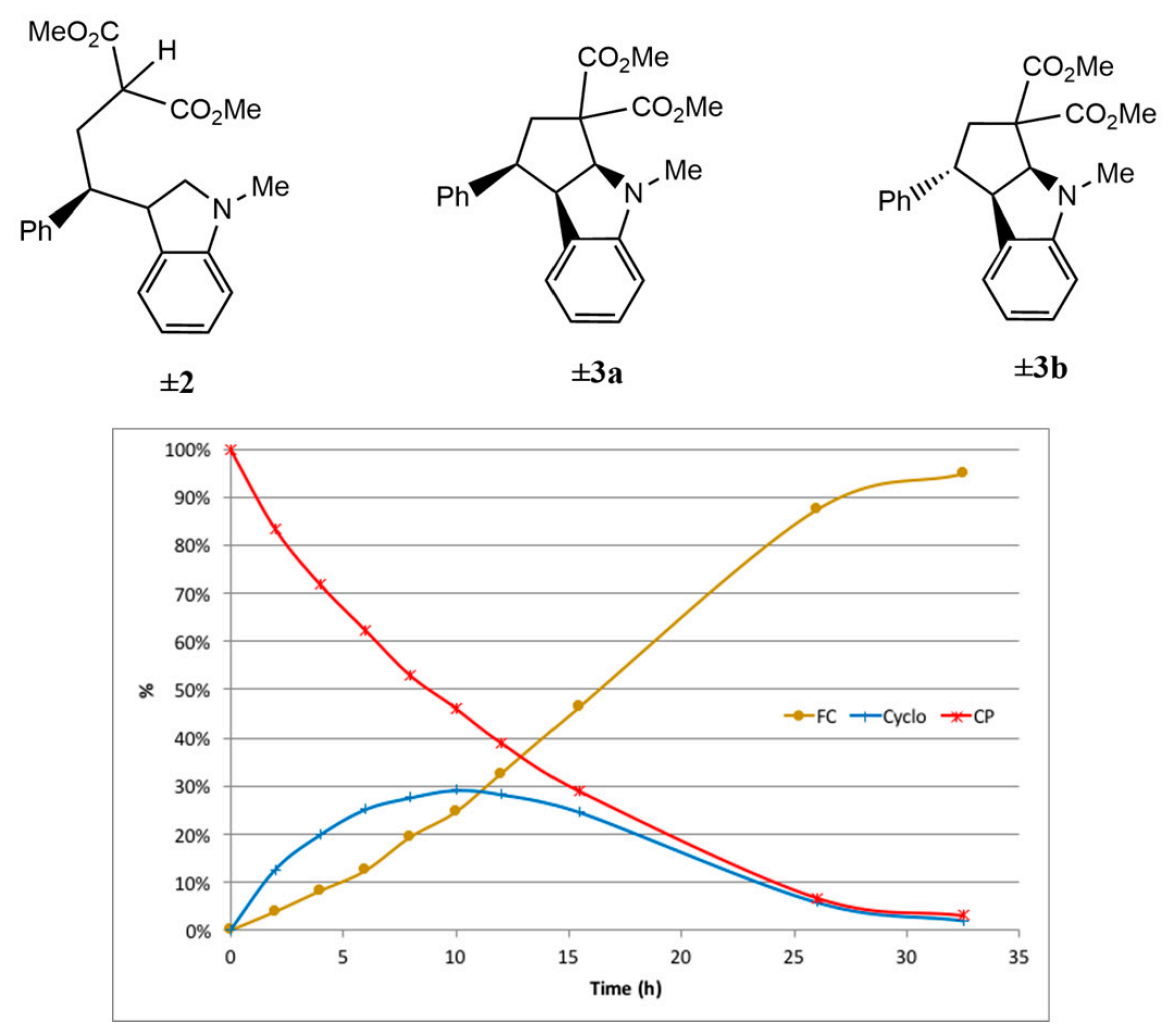
Conversion vs time; Friedel–Crafts (FC) product **2**, annulation (Cyclo) product **3a** + **3b**, 1,1-cyclopropane diester **1** (CP).

With a solid understanding of the NMR behavior of these species in hand, we proceeded to experimentally monitor the reaction (Figure 5). A mixture of cyclopropane **1** and N-methylindole (1.3 equiv.), in the presence of 10 mol% Ca(NTf_2_)_2_ and 10 mol% NBu_4_PF_6_ as a 1 M solution of *d8*-THF, was monitored by ^1^H NMR at 50 °C. We included the catalyst in this portion of our study because the reaction did not proceed at a measurable rate without the catalyst. During the initial stage of the reaction (Figure 5), the rate of annulation was faster than the Friedel–Crafts reaction, suggesting that the barrier to forming **3** was lower than that for **2** in THF. At approximately 54% conversion of **1**, **3** reaches its maximum concentration, then decreases while the concentration of **2** continues to increase. The reaction proceeds to the full conversion of **1** to **2**, which is in agreement with the computational results, which indicate that the barrier for the formation of the reactive intermediate is relatively low (30 kcal/mol; Table 4 and Figure 4), especially for the temperatures used in our experiments. IC1 (which goes on to form **2**) is ~8 kcal/mol more stable than IC2 (which forms **3;** Table 4); and, once formed, the barrier for IC1 converting to **2** is ~4 kcal/mol lower than the barrier for IC2 converting to **3** (Table 4). Finally, FC product **2** is 5–11 kcal/mol more stable than annulation product **3** (Table 3). These results are also in agreement with the results obtained previously by Dulin et al. [5].

## 3. Methods

### 3.1. Geometry Optimizations of Reactants, Diastereomeric Reactive Intermediates, Transition States and Products

The geometry optimizations of all stereoisomers of the reactive intermediate **4**, along with products **2** and **3**, were performed using the hybrid B3LYP functional with a 6-31G** basis set. In addition, we performed transition state searching to identify the barriers connecting the reactants to the lowest energy intermediate (IC1), connecting IC1 to FC product **2**, and connecting IC2 to annulation product **3**. Minima were confirmed as having no negative frequencies while the single negative frequencies associated with the transition states were animated and visualized to confirm that the barriers connected the reactant to IC1 and IC2, and IC1 and IC2 to their respective products. Previous work has shown DFT to be a computationally efficient and reliable approach for studying reaction mechanisms [15,17,20,21,22]. Studies by Wanapun et al. [17] have shown that the 6-31G** basis set has accurately described similar systems. All calculations were performed using the computational package Gaussian16 [23]. The IEFPCM implicit solvation model was used to perform calculations in solvents of varying polarities: toluene, chloroform, dichloromethane (DCM), and tetrahydrofuran (THF) [24]. Transition state searches were performed in DCM and THF.

The original experimental study used solvents dichloroethane (DCE), cyclopentyl methyl ether (CPME), THF, hexane, toluene, ethyl acetate, and 2-methyltetrahydrofuran (2-MeTHF). Except for DCE, these solvents are commonly used in organic reactions and represent a wide range of polarities. CPME and 2-MeTHF are green solvents (corn-derived) and are on industry approved lists. For our computational study, we determined that performing simulations in 4 different solvents with dielectrics ranging from 2.4 (toluene) to 9.1 (DCM) gave us enough breadth in solvent properties to model the experimental behavior. For instance, 2-MeTHF (ε = 7.0) was the solvent used in the original experimental study; however, the Gaussian software did not contain parameterized models for this solvent, so we used THF as a substitute (ε = 7.0). We did not use DCE (ε = 10.4) due to similar solvent properties with DCM (ε = 9.1). We did not use CPME as the Gaussian software did not contain parameterized solvent models for CPME, and the dielectric for CPME (ε = 4.8) was the same as the dielectric for chloroform. In our modeling, we did not use ethyl acetate due to its similar dissolution properties as chloroform, and we did not use hexanes because, experimentally, the reaction did not proceed in that solvent. 

The four stereoisomers of the proposed zwitterionic reactive intermediate **4** are shown in Figure 2. The first *R* or *S* designation in the nomenclature refers to the chirality of the **C3** atom from the cyclopropane, and the second designation describes the chirality of the **C4** chiral atom from methylindole. For each stereoisomer, an exhaustive, manual search for all low-lying minimum energy conformers was performed by varying the torsion angle about the **C2**–**C3** and **C4**–**C5** bonds of the reactive intermediate **4** (Figure 2). These bonds were sampled between −180° and 180° in 60° increments. An incremental change of 60° was sufficient for a complete sampling of the torsional space as test calculations using increments of smaller torsion angles did not identify any new structures. Not all initial torsional conformations survived geometry optimization (i.e., some initial structures collapsed to the same local minimum geometry). These will be fully discussed in the results section.

### 3.2. NMR Calculations

The ^1^H NMR spectrum associated with each local minimum energy conformation of the stereoisomers of **4** was calculated using gauge-independent atomic orbital (GIAO) theory. The chemical shifts are computed based on the response of the internal molecular nuclear magnetic field to an applied external magnetic field. Computationally, the external magnetic field affects the kinetic energy term in the Hamiltonian such that an origin, called the gauge origin, must be defined for the system [25]. GIAO is one method to define the gauge origin and uses the center of each atom as its own gauge origin [19]. This is a computationally efficient method that provides an accurate prediction of ^1^H NMR spectra [18,19]. The calculated ^1^H NMR spectra were obtained using the mixed GIAO model at the B3LYP level of theory with a 6-31G** basis set.

## 4. Conclusions

This study offers a detailed conformational and energetic rationale for the observed product distribution in the reaction of a DA cyclopropane with 1-methylindole, highlighting how subtle differences in the geometry of a transient zwitterionic intermediate can direct the reaction towards either Friedel–Crafts addition or annulation products. Our approach, combining density functional theory (DFT) calculations with experimental ^1^H NMR time studies, yielded a detailed understanding of the conformational mechanism.

Zwitterionic intermediate **4** can exist in several distinct stereoisomeric and conformational forms. We identified three principal intermediate conformer groups (IC1, IC2, and IC3). The IC1 conformer was consistently found to be the lowest in energy, particularly in polar solvents such as DCM and THF. Crucially, the geometry of IC1, characterized by a shorter C1_anion_–H4 distance (e.g., ~2.35–2.38 Å in DCM), predisposes it towards hydrogen abstraction, leading to the formation of the Friedel–Crafts product **2**. Conversely, the IC2 conformer, though generally higher in energy (approx. 3–8 kcal/mol), exhibited shorter C1_anion_–C5 distances (e.g., ~3.24 Å for the SR stereoisomer in DCM), making it the most likely precursor for the annulation reaction to form product **3**. The IC3 conformers, with larger distances between the reactive C1_anion_ and both H4 and C5, were deemed less likely to proceed to either product.

Thermodynamic calculations of the products indicated that Friedel–Crafts product **2** was significantly more stable (by approximately 5–7 kcal/mol in DCM) than annulation products **3a** and **3b**. These computational predictions were corroborated by ^1^H NMR time studies conducted in *d8*-THF. Experimentally, annulation product **3** was observed to initially form faster, reaching a maximum concentration before gradually converting to the more thermodynamically stable Friedel–Crafts product **2**, which was the predominant experimental product upon reaction completion. This observation aligns with the calculated relative stabilities, suggesting a kinetically controlled formation of **3** and a thermodynamically controlled outcome favoring **2**. Furthermore, the calculated ^1^H NMR chemical shifts for the proposed intermediate **4**, particularly the downfield shift in H5, supported the presence of a partial positive charge on C5, consistent with the zwitterionic nature of the intermediate.

## Figures and Tables

**Table 1:** Reactive intermediate conformers for the RS/SR enantiomer of **4** in CH_2_Cl_2_ computed using B3LYP/6-31G** in IEFPCM solvent. H atoms were removed from the images of IC1-IC3, for clarity. Atoms colored in teal highlight the torsion angles that were sampled to interconvert between various conformations of **4**.

Intermediate Conformer 1 (IC1)	Intermediate Conformer 2 (IC2)	Intermediate Conformer 3 (IC3)
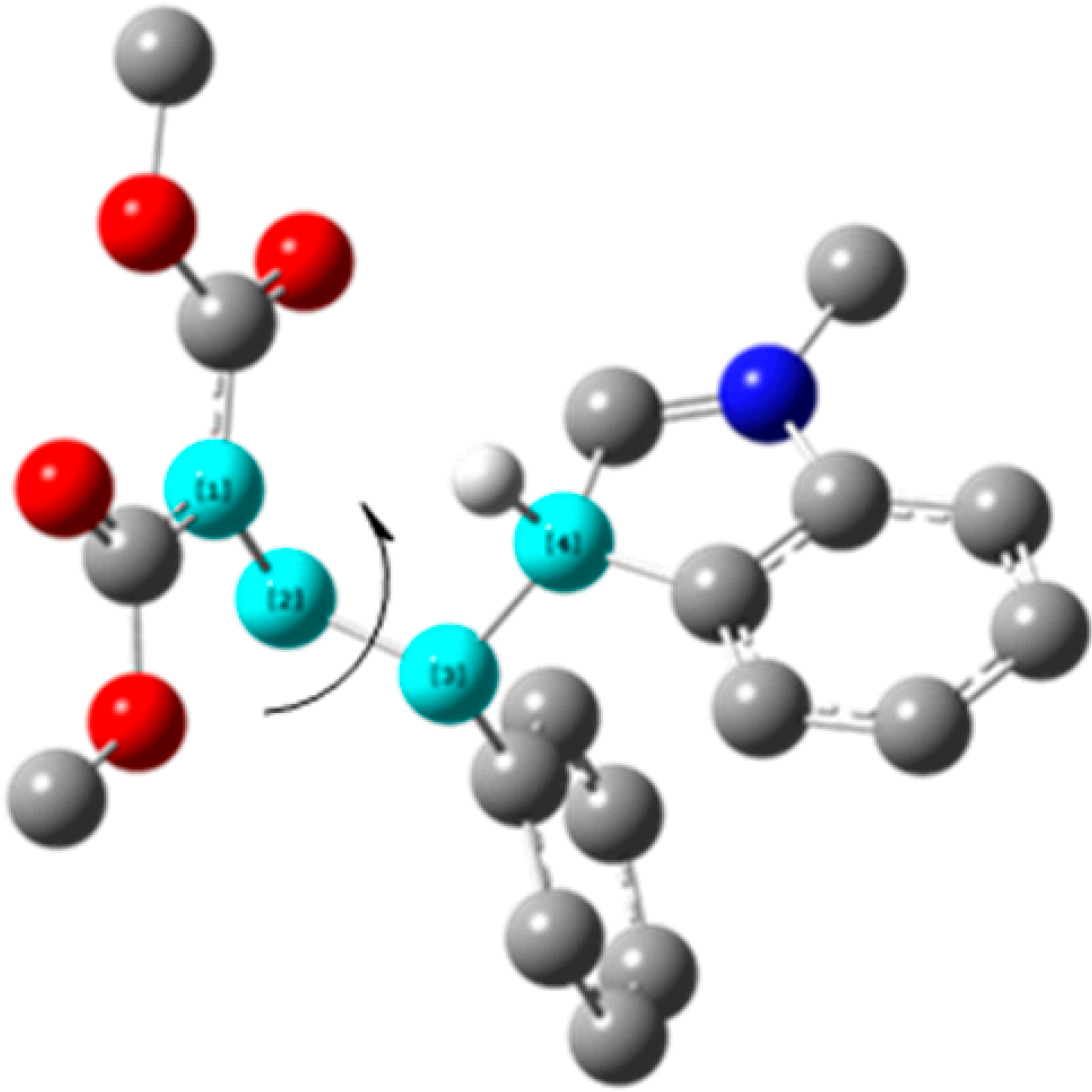	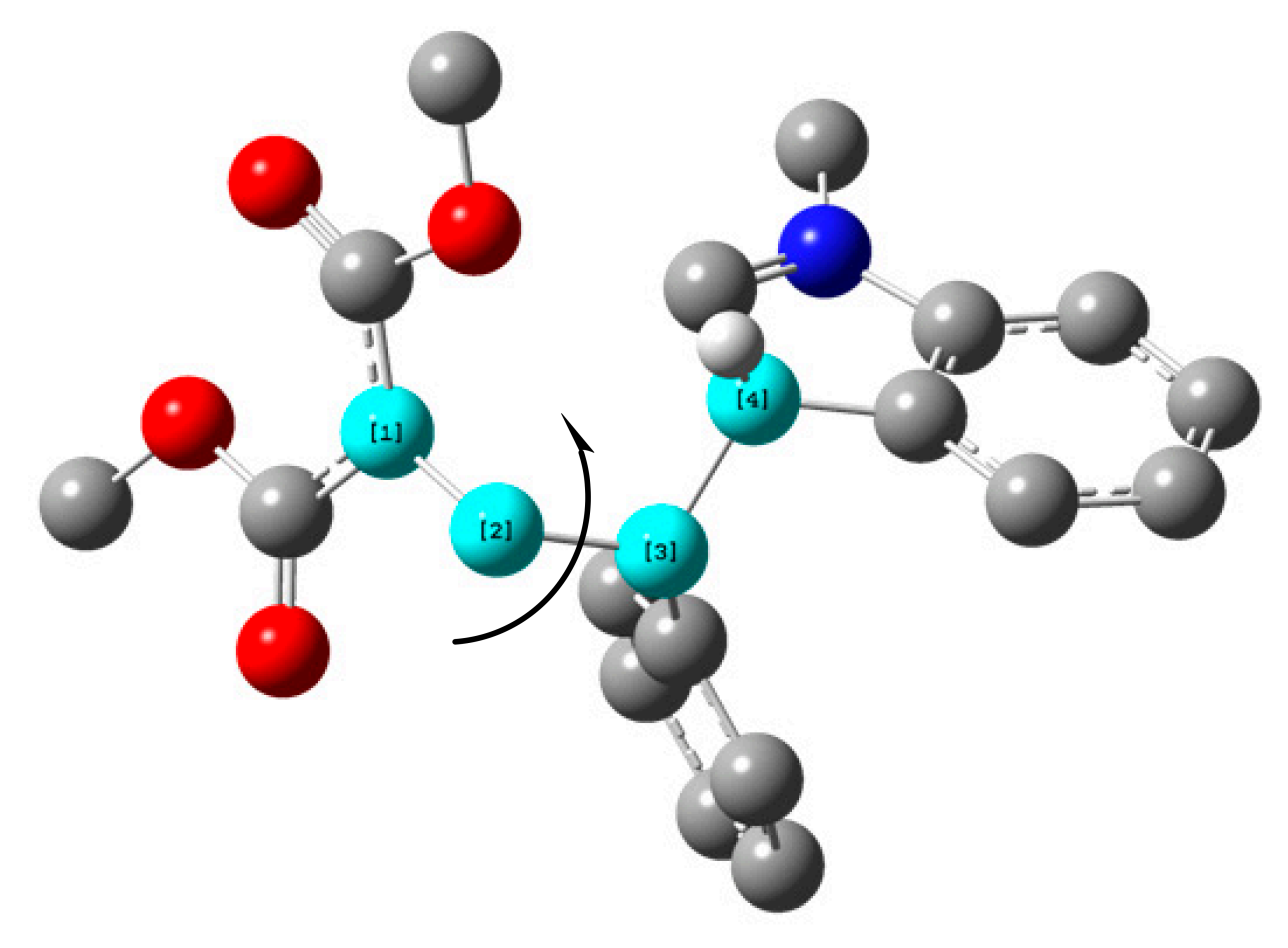	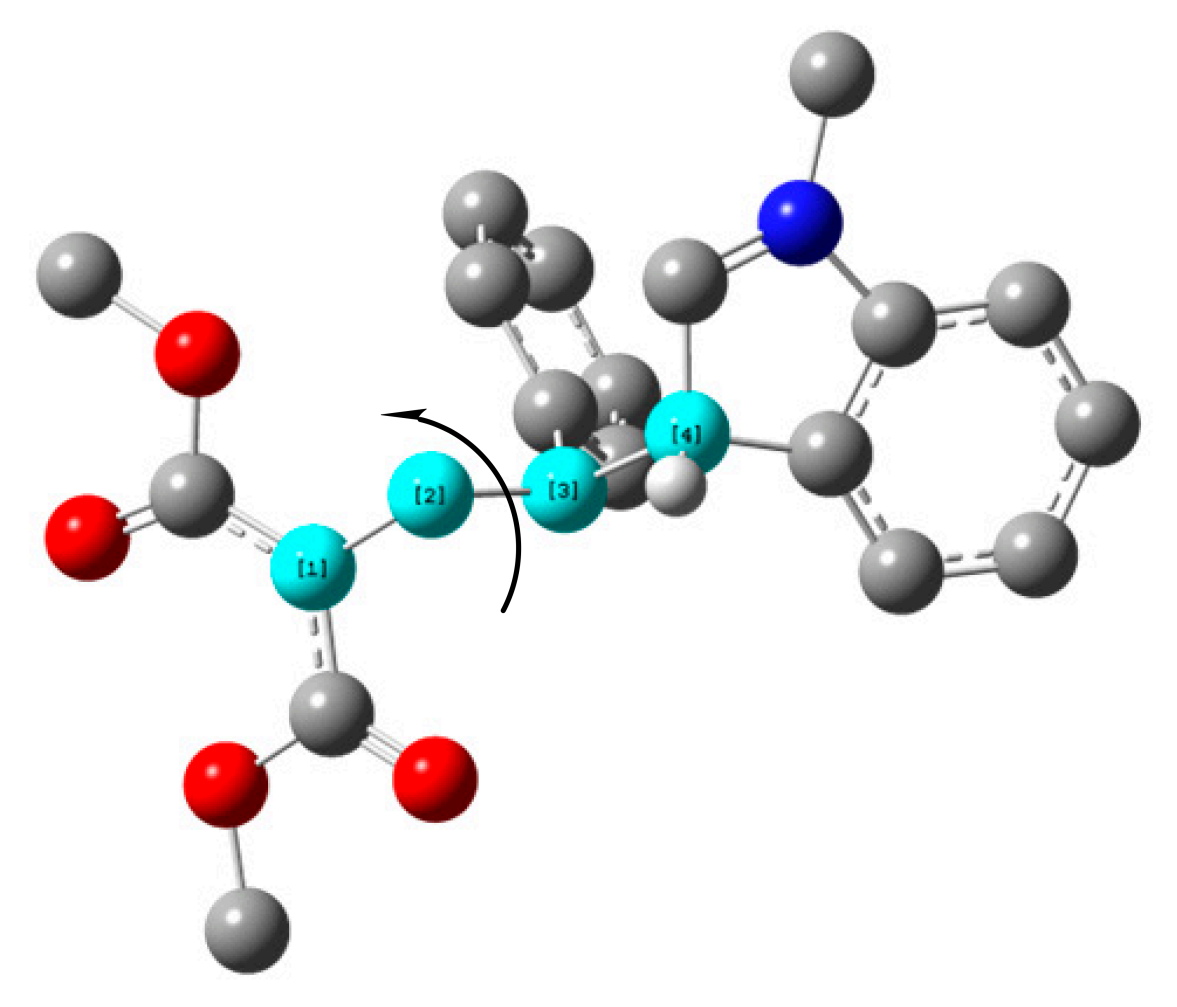
C1–C2 –C3–C4 dihedral		
±50°	±64°	±180°

**Table 2:** Relative reactive intermediate enthalpies (kcal/mol) and intramolecular distances (Å) of intermediate conformers (IC1–IC3) of **4** computed using B3LYP/6-31G** in IEFPCM solvent.

Conformer	Stereo- Isomer	E relative to Lowest E Diastereomer Within Same Solvent	E relative to Lowest E Structure in Any Solvent	C1_anion_–H4 Distance	C1_anion_–C5 Distance
***dichloromethane (ε = 9.1)***					
1	RS/SR	0.00	0.00	2.38	3.58
1	RR/SS	2.64		2.35	4.24
2	RS/SR	7.84		3.38	3.24
2	RR/SS	5.41		4.26	3.70
3	RS/SR	7.75		4.03	4.60
3	RR/SS	7.30		4.74	4.53
***tetrahydrofuran (ε = 7.5)***					
1	RS/SR	0.00	0.72	2.37	3.57
1	RR/SS	2.73		2.25	4.15
2	RS/SR	8.01		3.37	3.23
2	RR/SS	5.51		4.26	3.70
3	RS/SR	8.11		4.03	4.60
3	RR/SS	7.66		4.74	4.52
***chloroform (ε = 4.8)***					
1	RS/SR	0.00	2.99	2.32	3.56
1	RR/SS	3.94		2.18	4.11
2	RS/SR	8.52		3.35	3.18
2	RR/SS	5.76		4.25	3.69
3	RS/SR	9.26		4.02	4.59
3	RR/SS	8.79		4.73	4.51
***toluene (ε = 2.4)***					
1	RS/SR	0.00	7.84	2.20	3.53
1	RR/SS	3.45		2.08	4.06
2	RS/SR	9.31		3.34	3.02
2	RR/SS	6.20		4.21	3.64
3	RS/SR	11.81		3.99	4.58
3	RR/SS	11.23		4.71	4.49

**Table 3:** Product enthalpies (kcal/mol) computed using B3LYP/6-31G** in IEFPCM solvent.

Product	ΔE DCM	Tol	CLF	THF
**2**	0.00	0.00	0.00	0.00
**3a (cis)**	5.29	4.65	4.24	5.23
**3b (trans)**	7.43	6.85	6.40	7.37

**Table 4:** Relative enthalpies (ΔE, kcal/mol) for stationary points along the reaction path from 1,1-cyclopropanediester **1** + 1-methylindole to form the Freidel–Crafts **2** and annulation products **3a** in DCM and THF. Energies are shown relative to reactant energies computed with B3LYP/6-31G** in IEFPCM solvent.

Structure	ΔE (DCM)	ΔE (THF)
Reactants	0.0	0.0
TS (reactants—IC1 (SR))	30.0	30.6
IC1 (SR)	12.9	13.6
IC2 (SR)	20.7	21.6
TS (IC1 (SR)—FC (2))	18.2	18.8
TS (IC2 (SR)—annulation (3b))	22.6	23.3
FC product (2)	−22.3	−22.0
annulation product (3b)	−14.9	−14.6

**Table 5:** Predicted chemical shifts for H4 and H5 in THF for **2**, **3** and **4,** calculated using the GIAO model with the B3LYP/6-31G** level of theory with the IEFPCM solvent model for THF.

Species	Conformer	Stereoisomer	H4	H5
**2**			3.86	7.23
**3a**		±cis	3.85	4.94
**3b**		±trans	4.17	4.66
**4**	IC1	RR/SS	6.59	8.68
		RS/SR	5.59	11.37
	IC3	RR/SS	4.52	8.72
		RS/SR	4.61	8.95
	IC2	RR/SS	4.07	8.86
		RS/SR	5.08	9.48

## Data Availability

The original contributions presented in this study are included in the article/Supplementary Material. Further inquiries can be directed to the corresponding author.

## Supplementary Materials

The following supporting information can be downloaded at https://www.mdpi.com/article/10.3390/molecules31214023/s1, Figure S1: Relative energies and C1_anion_-H4 and C1_anion_-C5 distances for IC1, IC2 and IC3; Figure S2: Structures of intermediate **4** and products **2** and **3** with mechanistically important hydrogens labeled.
