# Supplementary material for: Thermodynamic Insight into Donor–Acceptor Cyclopropane Reactions with 1-Methylindole
Source: Molecules. 2026 Nov 8;31(21):4023. doi: 10.3390/molecules31214023 (PMC13765313; doi:10.3390/molecules31214023)
Supplement: Supplementary file 1 [file molecules-31-04023-s001.zip › molecules-4536960-supplementary.pdf]

Mechanistic Insight into Donor-Acceptor Cyclopropane Reactions with 1-Methylindole

Charles Biaku, Boyi Zhang, Parker Maloney, Malik Bell, Kristine Nolin and Carol A. Parish*

Department of Chemistry, Gottwald Center for the Sciences, University of Richmond, 28 Westhampton Way, Richmond, VA 23173

Supplemental Information

Table of Contents

Figure S1. Relative Energies and $C1_{anion}$-H4 and $C1_{anion}$-C5 distances for IC1, IC2 and IC3.

Figure S2. Structures of intermediate **4** and products **2** and **3** with mechanistically important hydrogens labeled.

Figure S1. Relative Energies and C1$_{anion}$-H4 and C1$_{anion}$-C5 distances for IC1, IC2 and IC3.

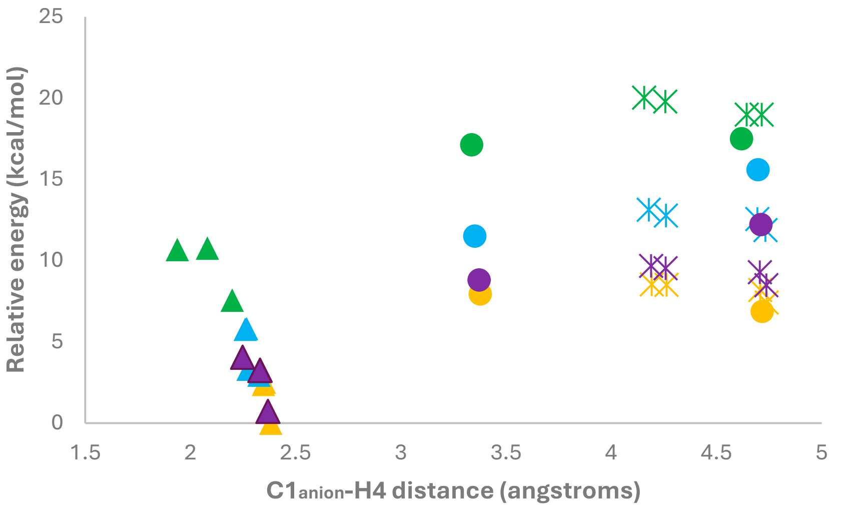

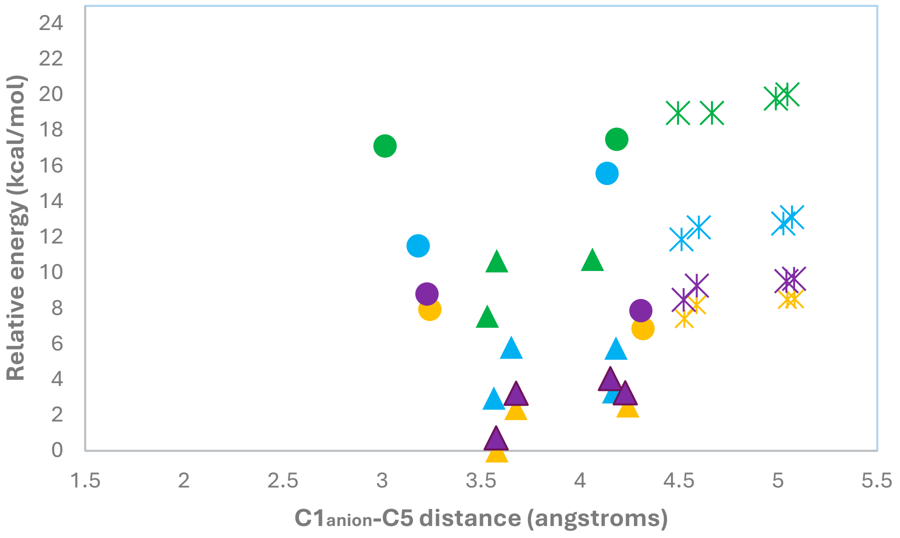

Relative energies (kcal/mol) of IC1 (triangles), IC2 (circles) and IC3 (stars) relative to ***C1$_{anion}$–H4*** *and* ***C1$_{anion}$–C5*** *distances (Å) for a variety of solvents of varying polarity DCM (orange), THF (purple), chloroform (blue), toluene (green).*

Figure S2. Structure of intermediate **4** and products **2** and **3**, with mechanistically important hydrogens labeled.

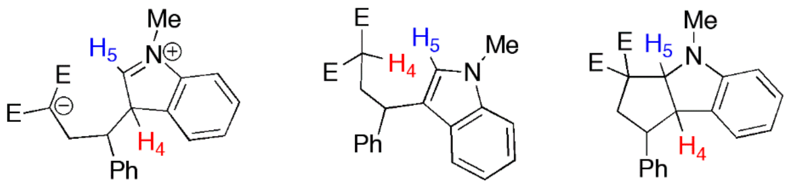

4 **2** **3**

intermediate conformations

products
